# Targeting Optimal Bone Regions: Correlations Between Bone Density and DNA Quality in Small Skeletal Elements

**DOI:** 10.3390/genes16030291

**Published:** 2025-02-27

**Authors:** Živa Miriam Geršak, Vladka Salapura, Eva Podovšovnik, Irena Zupanič-Pajnič

**Affiliations:** 1 Clinical Institute of Radiology, University Medical Centre Ljubljana, Zaloška 7, 1000 Ljubljana, Slovenia; 2 Faculty of Tourism Studies-Turistica, University of Primorska, Obala 11a, 6320 Portorož, Slovenia; 3 Institute of Forensic Medicine, Faculty of Medicine, University of Ljubljana, Korytkova 2, 1000 Ljubljana, Slovenia

**Keywords:** DSCT, DNA, WWII, bone density, skeletal remains, genetic identification

## Abstract

**Background**: Identifying the optimal bone regions for DNA analysis is critical, as DNA preservation and quality vary significantly across bone types and structures and is defined as intra-bone variability. This study aimed to evaluate the correlation between computed tomography (CT)-measured bone density and DNA preservation in small skeletal elements to identify optimal regions for DNA analysis. **Methods:** 137 bones from six skeletal elements excavated from a single burial site were analysed using Dual-Source CT (DSCT) to map compact and cancellous regions. DNA was extracted using a demineralisation method and quantified via real-time PCR to assess DNA quantity and degradation. **Results:** Among 461 bone regions analysed (137 bones; patellae, calcaneus, talus, the navicular bones, the cuboid bone, and the medial cuneiform bone), a significant difference in DNA quantity was observed only in the calcaneus, where the sulcus contained more DNA than the body. No significant differences in the degradation index were detected among bone segments or skeletal elements. Correlations between CT-measured bone density and DNA quantity or degradation index were region-specific. Higher bone density correlated positively with DNA quantity in compact regions of the calcaneus and talus. Regarding degradation, a positive correlation (higher bone density → higher degradation) was observed in the patella’s anterior surface, while a negative correlation (higher bone density → lower degradation) was found in the talus’s sulcus, the opposite side of the talar sulcus, the posterior calcaneal articular facet, and the cuboid’s tuberosity. No significant correlations were found in other bone segments. **Conclusions:** Our study identified small skeletal elements, particularly the patella and the navicular bone, as promising sources for DNA analysis. While bone density correlated with DNA preservation in some cases, the relationship was inconsistent. Our findings support the use of small bones in forensic and archaeological research and warrant further investigation.

## 1. Introduction

DNA analysis from skeletal remains has become a cornerstone in forensic science, anthropology, and archaeology, offering profound insights into human identity, ancestry, and population history. However, the success of DNA recovery and analysis is heavily dependent on the preservation state of the skeletal material. The success of DNA recovery and analysis depends heavily on the preservation state of the skeletal material, which is influenced by intrinsic factors, such as bone density and structure, as well as extrinsic factors, including environmental conditions and post-mortem degradation [1,2,3,4,5,6,7,8,9,10]. Recent advancements in DNA extraction and analysis methods have improved our ability to work with degraded remains; yet, identifying skeletal regions that consistently yield high-quality DNA remains a significant challenge [3,5,11,12,13,14,15,16,17].

Previous studies have established that the petrous portion of the temporal bone yields the highest DNA quantities among human skeletal elements, attributed to its dense, compact structure and low remodelling rate, which protect DNA from environmental factors [18,19,20,21,22,23,24].

Several recent studies on bones from the World War II period (including metatarsals, metacarpals, ribs, vertebrae, and femurs) further demonstrate that DNA quantity and preservation depend on the sampling site and the selection of trabecular or compact bone tissue [25,26,27,28,29,30,31]. For example, ribs exhibited higher DNA concentrations in their trabecular regions (proximal and distal parts) [28]. Similarly, in metatarsals and metacarpals, more DNA was recovered from the epiphyses compared to the diaphysis, while in vertebrae, the vertebral arch yielded higher DNA concentrations [25,27]. No significant difference was observed in femurs between the epiphysis and diaphysis, likely due to this long bone’s thick, compact cortical bone layer. Notably, femurs provided higher DNA yields from their diaphysis than the diaphysis of metatarsals and metacarpals, likely due to better preservation of the thick cortical bone in femurs [25].

When processed with advanced extraction techniques, trabecular-rich bones such as epiphyses have demonstrated promising DNA yields comparable to compact elements like femurs. This is likely due to the retention of soft tissue remnants in their intertrabecular spaces, which enhances DNA preservation in cancellous-rich regions [25,26,27,28,29,32,33,34]. Additionally, sesamoid bones, such as patellae, exhibit high DNA recovery due to their mixed compact and trabecular composition [30]. The bones included in our study have not yet been thoroughly examined for their internal structure. Unlike long bones, where the diaphysis is made of compact bone and the epiphyses are composed of cancellous bone, the inner structure of smaller bones is not as straightforward. Sesamoid bones, for example, typically have a thin layer of cortical bone and are primarily composed of cancellous bone.

To identify optimal DNA sampling sites, researchers have employed tools such as Attenuated Total Reflectance (ATR), Fourier Transform Infrared (FTIR) spectroscopy, and computed tomography (CT) techniques, including micro-CT [7,35,36]. ATR-FTIR provides insights into the chemical composition of bone, analysing surface layers to a few micrometres’ depth. Micro-CT offers high-resolution visualisation of bone microstructure, while CT enables whole-bone analysis and is widely used to measure bone density in living populations. Among these, Dual-Source CT is promising for accurately analysing bone density in forensic and archaeological contexts [37,38,39,40].

Despite these advancements, the extent to which bone density—measured via CT—correlates with DNA preservation and degradation across diverse skeletal elements remains poorly understood. While trabecular-rich bones may yield higher quantities of DNA in specific scenarios, their susceptibility to degradation has not been fully characterised. Furthermore, most studies focus on elements like the petrous bone, leaving gaps in our understanding of intra-bone variability and DNA preservation in smaller skeletal elements [21,29,32].

This study aimed to evaluate the correlation between CT-measured bone density and DNA preservation in small skeletal elements to identify optimal regions for DNA analysis. We compared the amount of DNA and the degradation index among bone segments and structures. The correlation between the amount of DNA and measured bone density and between the degradation index and measured bone density among bone segments and different bone structures was investigated.

## 2. Materials and Methods

### 2.1. Sample Selection, CT Imaging, and Region of Interest Selection

The study included 137 well-preserved whole bones from six different skeletal elements: 15 patellae, 26 calcanei, 12 tali, 34 navicular bones, 29 cuboid bones, and 21 medial cuneiform bones. These bones were excavated from the Konfin Shaft II Mass Grave [25], where prisoners of war were executed and dumped in June 1945. Discovered in 2007, the remains were mixed in a pile under large rocks, stones, tree trunks, and 50–120 cm of soil. The shaft maintained a constant temperature of 5–10 °C. The bones were scattered in multiple layers, with the highest density near the entrance. The upper layers were damaged due to dynamite, but some long bones, pelvis, and parts of the spine and ribs allowed partial individualisation. Well-preserved bones were found in pockets and on the stone base of the shaft. The victim count was determined by analysing long bones, hip bones, collarbones, and specific bones like the sacrum and sternum handles. Fewer small hand and foot bones were found, likely lost due to water runoff, environmental exposure, or animal activity. Various factors affect DNA preservation, including the preservation conditions, soil composition, burial depth, temperature, and other environmental influences, as well as differences in storage methods after excavation and the time elapsed between excavation and DNA analysis [11,32,41,42,43,44,45,46]. However, in this study, all bones were excavated from the same location and similar depths, stored using identical methods, and analysed after the same time interval, thereby controlling for these variables. This enabled us to compare the bones of different skeletons as they were subjected to the same decomposition and environmental factors for the same amount of time.

A total of 137 bones were analysed using a technologically high-performance DSCT device (Siemens Definition FORCE, Siemens, AG). The imaging protocol was explicitly adapted to highlight the skeletal structure of small bones. This tailored protocol and region of interest (ROI) selection are described in detail in Geršak et al. [47]. Each skeletal element was divided into smaller parts that were large enough to enable sampling for genetic identification according to established DNA extraction protocols [12].

The patella was divided into the apex, anterior, and posterior surface; the calcaneus was divided into four parts, including its posterior process, the body, the sulcus, and the anterior process; the talus was divided into five parts, including the head, talar sulcus, the area on the opposite side of talar sulcus, trochlea, and posterior calcaneal articular facet; the navicular bone was divided into its proximal articular surface, distal articular surface, and tuberosity; the cuboid bone was divided into its proximal articular surface, tuberosity, and distal articular surface; and finally, the medial cuneiform bone was divided into its proximal articular surface, medial surface, and distal articular surface.

The CT-measured density and subsequent DNA analysis were performed on the same bone fragment. Prior to CT scanning, only the surface of the bones was gently cleaned to remove dirt and small rocks, avoiding damage to delicate elements. Bones were placed on sterilised covers (used in surgery) and handled with rubber gloves, which were changed between each scan. After CT scanning, the bones were cleaned and prepared for further DNA analysis following the protocol outlined by Zupanič Pajnič [12].

The study was approved by The National Medical Ethics Committee of the Republic of Slovenia (0120-233/2020/3).

### 2.2. Sample Preparation

All 137 bones underwent meticulous cleaning to remove surface contaminants. The process began with mechanical cleaning using a rotary sanding tool (Schick, Schemmerhofen, Germany), followed by chemical cleaning with 5% Alconox detergent (Sigma-Aldrich, St. Louis, MO, USA). The bones were then rinsed with sterile bi-distilled water and 80% ethanol and allowed to air-dry overnight. Each bone was carefully sown into parts with a sterilised diamond saw (Schick, Schemmerhofen, Germany), as the obtained CT images and bone density evaluation outlined.

All 461 bone parts were processed into fine bone powder using a Bead Beater MillMix 20 homogeniser (Tehtnica, Domel, Železniki, Slovenia). Grinding was performed for 1 min at a frequency of 30 Hz in metal vials pre-cooled with liquid nitrogen to prevent overheating. The bone fragments were also cooled before grinding.

### 2.3. DNA Extraction, Purification, and Quantification

DNA was extracted from 0.5 g of bone powder using a full demineralisation protocol outlined by Zupanič Pajnič [12]. In one of our recent studies [29], we compared DNA extraction protocols for trabecular and compact bone using full dissolution (FD) and partial dissolution (PD). Our findings were that in compact bone, FD yields significantly more DNA than PD. In trabecular bone, there is no statistically significant difference between the FD and PD methods. The extracted DNA was purified and eluted in 50 μL of TE buffer using the EZ1 Advanced XL system (Qiagen, Hilden, Germany) and the EZ1 & EZ2 DNA Investigator Kit (Qiagen), following the manufacturer’s instructions.

A real-time PCR (qPCR) method with the PowerQuant System (Promega, Madison, WI, USA) was used to evaluate DNA content and degradation. This system quantified total autosomal and male DNA while detecting potential PCR inhibitors. DNA degradation was assessed by amplifying two targets of different lengths: a short Auto target (85 bp) and a long Deg target (294 bp). The Auto/Deg ratio, calculated with the PowerQuant Analysis Tool (Promega), measured DNA degradation. All samples were quantified in duplicate as per the manufacturer’s guidelines. Amplification reactions were carried out using the Quant Studio 5 Real-Time PCR system (Applied Biosystems, Foster City, CA, USA). Threshold values for the IPC shift and the Auto/Deg ratio were set at 0.30 and 2, respectively, in line with the manufacturer’s recommendations [48]. The DNA concentration for each bone sample was calculated in ng DNA per gram of bone.

### 2.4. STR Typing

For kinship analysis in forensic and archaeological investigations and for identification through comparison with personal items of the deceased in forensic investigations, successful autosomal STR genetic typing is essential [49,50]. Eighteen samples were chosen for autosomal STR typing and the obtained genetic profiles were used to confirm DNA authenticity by comparison with elimination database profiles. Since the femurs from all Konfin II grave victims were already typed for autosomal STRs [27], profiles from this study were compared to check for matching endogenous bone DNA (authentic bone DNA and not contamination of modern DNA). In selecting samples, we included every skeletal element—two cuboids, two patellae, one talus, two medial cuneiforms, three calcanei, and three navicular bones. For certain elements, we analysed multiple parts of the same bone (two sections for the cuboid, two for the patella, three for the talus, and two for the medial cuneiform) to verify whether different regions yield identical genetic profiles, as expected. A complete list of the bones and their sections is provided in a table within SM3.

### 2.5. Contamination Control Measures and Authenticity Criteria

Strict precautions were implemented while handling ancient bones to prevent and monitor contamination with modern DNA. Cleaning and grinding were performed in a dedicated facility exclusively designed for processing ancient skeletons within a microbiological safety hood (Iskra Pio, Šentjernej, Slovenia). All work surfaces, equipment, and the biological safety cabinet were thoroughly disinfected using a bleach, water, and ethanol protocol, followed by overnight UV irradiation. This stringent sanitation procedure was applied to all reagents, laboratory plastics, and equipment, which were sterilised and UV-irradiated. Each sample was processed with clean equipment, and extraction-negative controls (ENCs) were included in each extraction batch. Negative controls were also incorporated into every qPCR reaction to ensure the purity of reagents and plastics. Additionally, an elimination database was created to track possible contamination with modern DNA.

### 2.6. Statistical Analysis

All bone density values in Hounsfield units (HU) from all selected ROIs and all DNA quantity values obtained from the analysed parts of the bone were included in statistical analysis.

Concerning the aim of the study, the following research questions were formulated:

Are there differences in the amount of DNA and the degradation index among bone segments of different skeletal elements?

Are there differences in the amount of DNA and the degradation index among different bone structures by skeletal element?

Are there correlations between the amount of DNA and measured bone density and between the degradation index and measured bone density among bone segments of different skeletal elements?

Are there correlations between the amount of DNA and measured bone density and between the degradation index and measured bone density among different bone structures by skeletal element?

The questions mentioned above were tested using 95% confidence intervals for means or medians [41], using bootstrapping [51,52] with 1000 samples. The correlation hypotheses were tested using Spearman’s correlational coefficients. All statistical analyses were performed using IBM SPSS Statistics, version 26.0.

There were undetermined values for the amount of the DNA (17 out of 461; 3.7%) and the degradation index (42 out of 461; 9.1%) in the database. For the amount of the DNA, undetermined values were substituted with the value 0,04, since the lowest amount of the detected DNA that was still useful for interpretation was 0,05. In the case of the degradation index, they were substituted with values according to the following formula:

$$Value\left(i\right)=Max\left(i\right)+SD(i)$$

where *i* stands for the skeletal element.

Descriptive statistics were calculated, and tests for the normality of the distribution of the amount of DNA were performed (see Supplementary Material, File S1). The Kolmogorov–Smirnov test was used to test the normality of the distribution. Given the abnormal data distribution, statistical differences were evaluated using 95% confidence intervals, with significance indicated by non-overlapping intervals. In tables, statistically significant correlations at the 0,01 level were marked with **, while statistically significant correlations at the 0.05 level were marked with * (see Supplementary Material, File S2).

## 3. Results

### 3.1. DNA Quantity and Degradation

Firstly, the DNA quantity and degradation were analysed. On average, the highest mean amount of DNA was extracted from the patella (M = 30.01; SD = 23.97), followed by the navicular bone (M = 24.42; SD = 23.68), the medial cuneiform bone (M = 22.79; SD = 19.79), the cuboid bone (M = 20.25; SD = 23.6), the talus (M = 13.61; SD = 20.1) and the calcaneus (M = 9.51; SD = 13.61). The highest extracted DNA was from the distal articular surface of navicular bone 16 (106.99 ng DNA/g bone).

**Figure 1:**
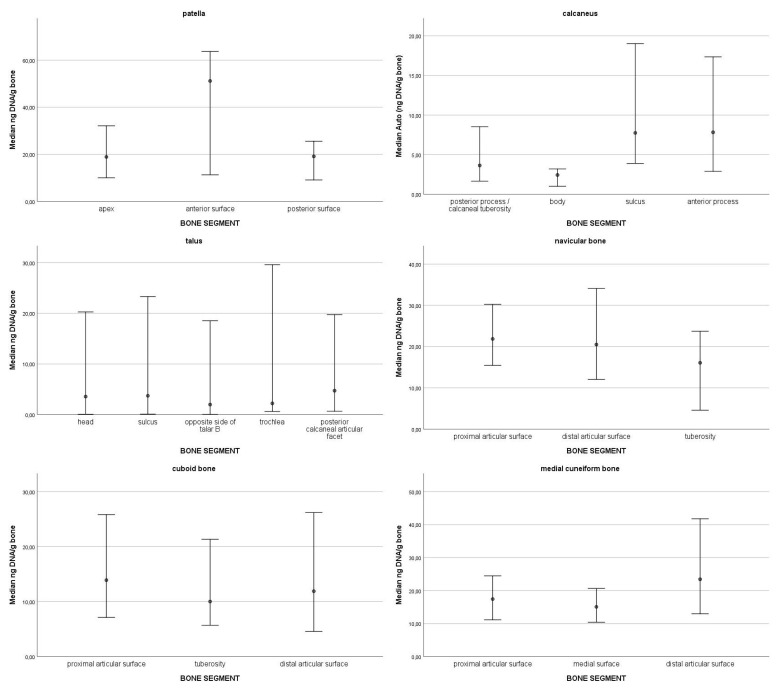
95% confidence intervals of medians for DNA quantity for each skeletal element.

**Figure 2:**
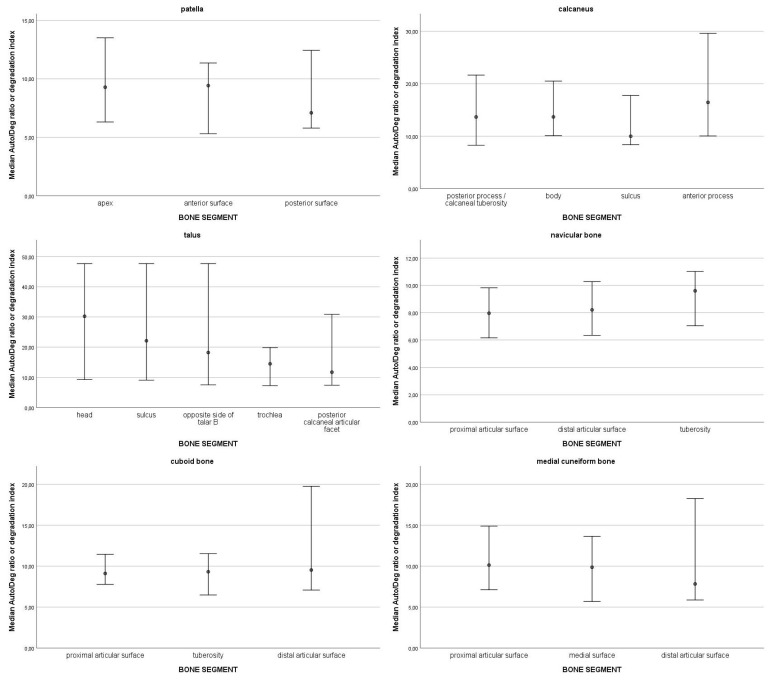
95% confidence intervals of medians for the degradation index by skeletal element.

**Figure 3:**
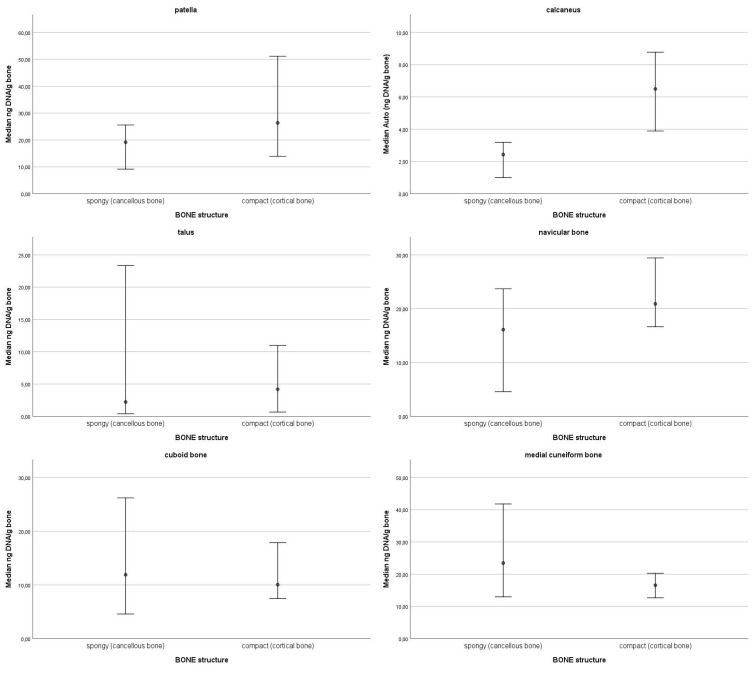
95% confidence intervals of medians for DNA quantity for each skeletal element by bone type.

**Figure 4:**
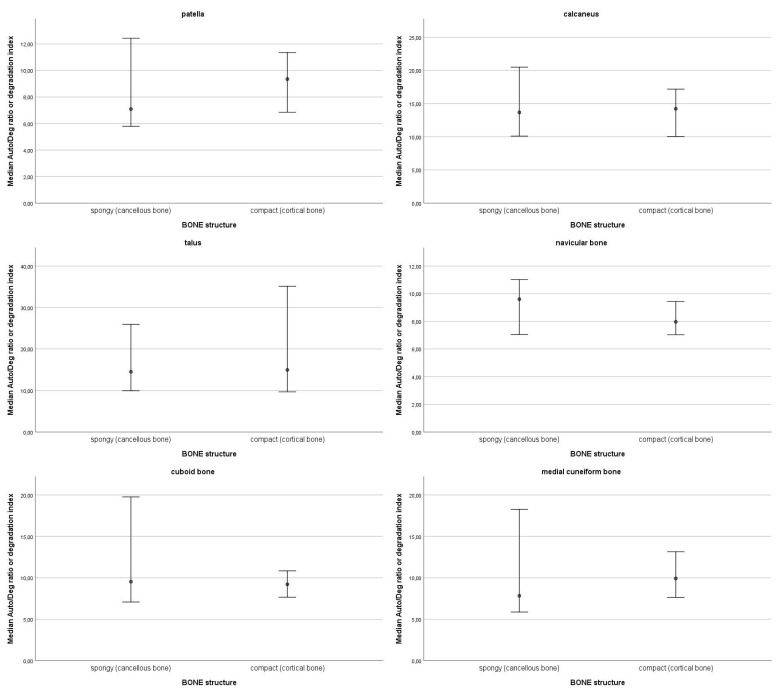
95% confidence intervals of medians for the degradation index by skeletal element by bone type.

In the top 10% of values of DNA quantity, most samples were from the navicular bone (13), followed by the patella (11), cuboid bone (10), medial cuneiform bone (7), talus (4), and lastly, calcaneus (1). The top 10% values ranged from 106.99 ng DNA/g bone to 50.43 ng DNA/g bone. The 95% confidence intervals of medians for DNA quantity for each skeletal element are presented in Figure 1. Statistically significant differences were observed only in one skeletal element, the calcaneus; there is significantly more DNA in the sulcus compared to the other parts of the bone. On average, the highest degradation index was observed in the talus (M = 22.99; SD = 16.6) and in the calcaneus (M = 22.37; SD = 21.11), followed by the cuboid bone (M = 13.24; SD = 12.34), the medial cuneiform bone (M = 11.91; SD = 9.01), the navicular bone (M = 10.92; SD = 7.55) and the patella (M = 10.12; SD = 6.43). The 95% confidence intervals of medians for the degradation index by skeletal element are presented in Figure 2. All 95% confidence intervals were overlapping. Therefore, no statistically significant differences in the degradation index were observed. DNA quantity and degradation index were also analysed by either cancellous or compact bone type. The 95% confidence intervals of medians for DNA quantity (Figure 3) and the degradation index (Figure 4) for each skeletal element are presented in their respective Figures. All observed 95% confidence intervals overlapped, showing no statistically significant differences. In most ENC samples, no Power Quant targets were detected. In cases where an amplification product was observed, the amount of DNA did not exceed the detection limit for the Power Quant kit, indicating no contamination (data available in Supplementary Material, File S2).

### 3.2. Correlations Between DNA Quantity and Bone Density

Spearman’s correlations among pairs of DNA quantity and bone density and degradation index and bone density for all bones were calculated. A statistically significant correlation was observed for the patella only on the anterior surface, where higher HU mean values corresponded to a higher degradation ratio (rho = 0.58; *p* = 0.02). A significant correlation was found in the anterior process for the calcaneus, with a higher HU mean linked to a higher DNA amount (rho = 0.49; *p* = 0.01). The talus showed multiple significant correlations. In the sulcus, a higher HU mean corresponded to a higher DNA amount (rho = 0.83; *p* < 0.01) and a lower degradation index (rho = −0.71; *p* < 0.01). On the opposite side of the talar sulcus, a higher HU mean was linked to a higher DNA amount (rho = 0.75; *p* < 0.01) and a lower degradation index (rho = −0.84; *p* < 0.01). In the trochlea, a higher HU mean correlated with a higher DNA amount (rho = 0.64; *p* = 0.03), while in the posterior calcaneal articular facet, it corresponded to a lower degradation index (rho = −0.64; *p* = 0.03). No significant correlations were found for the navicular bone or medial cuneiform bone. A significant correlation was observed in the tuberosity for the cuboid bone, where a higher HU mean corresponded to a lower degradation index (rho = −0.41; *p* = 0.03).

Lastly, correlations were calculated between DNA quantity and bone density, and degradation index and bone density for all bones according to bone structure. No statistically significant correlations were observed for the patella, navicular, cuboid, or medial cuneiform bone at the 0.05 level in either spongy or compact bone structures. For the calcaneus, a significant correlation was found in the compact bone, where higher HU mean values corresponded to a higher DNA amount (rho = 0.39; *p* < 0.01) and a lower degradation index (rho = −0.23; *p* < 0.05). No significant correlations were observed in the spongy bone. For the talus, a significant correlation was observed in the compact bone, with higher HU mean values linked to a higher DNA amount (rho = 0.64; *p* < 0.01) and a lower degradation index (rho = −0.66; *p* < 0.01). No significant correlations were found in the spongy bone. Detailed tables of all values and calculations for all correlations are compiled in Supplementary Material (Supplementary Materials, Files S1 and S2).

### 3.3. STR Typing

Eighteen samples were chosen for autosomal STR typing, and the obtained genetic profiles were compared to those of the femurs for authenticity and matching endogenous bone DNA. All 18 samples yielded full and complete profiles with all STR loci being amplified (17/17). The 18 samples belonged to 11 previously identified individuals (see table Supplementary Material, File S3). The matches were as follows. Six samples (two from the cuboid bone 3, three from talus 3 and one navicular bone 16) matched the same individual, previously identified through femur which was labelled as victim no. 50. Two samples from patella 2 matched the femur of the victim no. 24. Two samples of the medial cuneiform bone 8 matched the femur of the victim no. 54. The rest of the samples each belonged to a different individual; the medial cuneiform bone 5 to victim no. 17, the calcaneus 4 to victim no. 35, the cuboid bone 10 to victim no. 1, calcaneus 19 to victim no. 21, the calcaneus 22 to victim no. 26, the patella 13 to victim no. 57, the navicular bone 14 to victim no. 31, and the navicular bone 25 to victim no. 37. No samples matched the individuals involved in handling the samples that were included in the elimination database.

## 4. Discussion

Using DSCT, we measured bone density values and prepared targeted samples for DNA analysis based on their internal structure. Correlations between bone density, DNA quantity, and degradation index were analysed, revealing key findings.

A significant difference in DNA quantity was observed only in the calcaneus, where the sulcus contained more DNA than the body. There were no statistically significant differences in DNA quantity or the degradation index for all other skeletal elements. While the highest extracted DNA quantity was from the navicular bone’s compact area of distal articular surface extracted, the patellae had the highest mean DNA concentration. The talus and calcaneus showed the most DNA degradation, while patellae and the navicular bone had the lowest.

There were no statistically significant differences in the amount of DNA and the degradation index among different bone structures.

There are partial correlations between bone density (measured as HU mean values), DNA quantity, and degradation index across various skeletal elements. Higher HU values were associated with increased DNA amounts in regions such as the talus’s sulcus, its opposite side, the trochlea, and compact areas of the calcaneus and talus. A positive correlation (higher HU, higher degradation) was noted in the patella’s anterior surface for degradation. In contrast, a negative correlation (higher HU, lower degradation) was observed in the talus’s sulcus, its opposite side, the posterior calcaneal articular facet, and the cuboid bone’s tuberosity. However, these correlations were not consistent across all bone segments.

The results demonstrate the efficacy of autosomal STR typing, as all 18 samples produced complete genetic profiles (17/17 STR loci amplified). The genetic profiles matched those obtained from femur samples, confirming the authenticity and endogenous origin of the DNA. Importantly, no matches were found with the elimination database, underscoring the reliability of the sample handling procedures. Overall, these findings validate the use of small skeletal elements for forensic identification through STR profiling.

The findings of this study provide valuable insights into optimizing skeletal sampling strategies for forensic and archaeological DNA analysis. Although the petrous portion of the temporal bone is recognized as the highest-yielding skeletal element for DNA analysis due to its dense, compact structure and low remodeling rate [18,19,20,21,22,23,24], its sampling is often limited and infrequently used in forensic cases because destructive sampling precludes its regular use [23,53,54,55,56,57,58,59,60,61]. However, recent studies, including our own, demonstrate that small, cancellous-rich bones such as the patella and navicular can yield comparable or even superior DNA quantities. Our findings align with Andronowski et al. [35] who demonstrated that osteocyte lacunar density does not always predict DNA yield, as the trabecular bone’s retained soft tissue can enhance DNA preservation. This may explain why trabecular-rich bones (e.g., patella and navicular) yielded high DNA values, supporting their use as viable alternatives to the petrous bone in forensic and archaeological contexts [30,62,63]. This reinforces findings that trabecular-rich regions, such as the epiphyses of metatarsals and metacarpals, can provide substantial DNA quantities, challenging the conventional preference for cortical-rich long bones like the femur or tibia, which, despite their density, may not always yield optimal results. DNA yield is evidently more strongly influenced by bone microarchitecture and preservation conditions rather than cellular density alone. However, it is also true that trabecular bones degrade faster due to moisture absorption and microbial activity, potentially explaining the higher DNA degradation observed in the calcaneus and talus.

The DNA quantification was performed with qPCR using the PowerQuant System by Promega, which assesses the DNA degradation by amplifying two targets of different lengths: a short Auto target (85 bp) and a long Deg target (294 bp). As the DNA is degrading it is being split from longer fragments into shorter and shorter fragments. To simplify, the more the DNA is degraded, the higher quantities of short Auto targets there will be in the evaluated sample. The DNA quantity in the research conducted on non-forensic skeletal remains (meaning older than 50 years POI)—in other words, research conducted on historical (50–75 years POI) and ancient remains (hundreds to thousands of years POI)—the quantity of the Auto target is assessed, and that determines the interpretation of whether the DNA quantity is high or not. The effects on DNA degradation, however, are not—as proven by numerous studies—fully understood, and are only speculated based on environmental factors, diagenetic processes, and intrinsic bone properties such as density and microstructure. While factors like temperature, humidity, microbial activity, and soil composition are known to influence DNA preservation, their precise impact on degradation rates remains variable and case-dependent [11,32,41,42,43,44,45,46,64,65,66,67,68]. Additionally, differences in DNA fragmentation patterns across skeletal elements suggest that bone composition and postmortem conditions are crucial in determining DNA recoverability.

We opted for the use of DSCT for measuring the bone density as it can generate images of soft tissue or bone marrow by decomposing different tissue characteristics and is currently used for screening bone density with great diagnostic accuracy [37,38,39]. Micro-CT, in comparison, is an interesting method for investigating forensic implications, particularly for identifying individual-specific alterations or changes throughout historical periods [23,35,69,70,71,72,73,74,75]. While Micro-CT offers high-resolution imaging for forensic studies, its complexity, cost, and inability to analyse entire bones make DSCT a more practical approach.

For two skeletal elements, the sample size was limited (e.g., 12 talar bones, 15 patellae), however, overall trends remained clear. We opted not to slice bones blindly to differentiate compact and cancellous tissue, as this could have led to inconsistent cuts and DNA loss. Future research could expand sample sizes and explore newer DNA extraction protocols [17] that enable more precise sampling of compact bone areas within small skeletal elements or include non-autosomal DNA.

## 5. Conclusions

In conclusion, this study assessed DNA preservation in small skeletal elements alongside CT-measured bone density. Although trabecular-rich bones—such as the patella and navicular—tended to yield high DNA quantities, the association between bone density and DNA preservation was not consistently observed across all elements. From the perspective of DNA yield and the potential for complete profile generation, these small skeletal elements show remarkable promise. The findings suggest that the burial conditions or depositional environments may play a more significant role in DNA preservation and degradation than bone density alone. Future research could explore whether a correlation between bone density and DNA quantity exists in various burial contexts and across different post-mortem intervals, including ancient DNA samples.

## Data Availability

The original contributions presented in the study are included in the article/Supplementary Material, further inquiries can be directed to the corresponding author.

## Supplementary Materials

The following supporting information can be downloaded at: https://www.mdpi.com/article/10.3390/genes16030291/s1.
