# Supplementary material for: Targeting Optimal Bone Regions: Correlations Between Bone Density and DNA Quality in Small Skeletal Elements
Source: Genes (Basel). 2025 Feb 27;16(3):291. doi: 10.3390/genes16030291 (PMC11941782; doi:10.3390/genes16030291)
Supplement: Supplementary file 1 [file genes-16-00291-s001.zip › File S1.pdf]

Supplementary Material File S1

Descriptive statistics

Table Statistics Supplementary 1: Descriptive statistics and normality tests for the amount of DNA, by skeletal element.

		patella	calcaneus	talus	navicular bone	cuboid bone	medial cuneiform bone
N		45	104	60	102	87	63
Normal Parameters[a,b]	Mean	30,01	9,51	13,61	24,42	20,25	22,79
	Std. Deviation	23,97	13,61	20,10	23,86	23,60	19,79
Most Extreme Differences	Absolute	0,194	0,24	0,25	0,15	0,20	0,20
	Positive	0,194	0,23	0,23	0,15	0,19	0,20
	Negative	-0,114	-0,24	-0,25	-0,15	-0,20	-0,12
Test Statistic		0,194	0,24	0,25	0,15	0,20	0,20
Asymp. Sig. (2-tailed)		<0,01[c]	<0,01c	<0,01c	<0,01c	<0,01c	<0,01c
a. Test distribution is Normal.							
b. Calculated from data.							
c. Lilliefors Significance Correction.							

Table Statistics Supplementary 2: Descriptive statistics and normality tests for the degradation index, by sceletal element.

		patella	calcaneus	talus	navicular bone	cuboid bone	medial cuneiform bone
N		45	104	60	102	87	63
Normal Parameters[a,b]	Mean	10,12	22,37	22,99	10,92	13,24	11,91
	Std. Deviation	6,43	21,11	16,60	7,55	12,34	9,01
Most Extreme Differences	Absolute	0,20	0,24	0,21	0,21	0,25	0,18
	Positive	0,20	0,24	0,21	0,21	0,25	0,16
	Negative	-0,35	-0,18	-0,20	-0,14	-0,21	-0,18
Test Statistic		0,20	0,24	0,21	0,21	0,25	0,18
Asymp. Sig. (2-tailed)		<0,01[c]	<0,01c	<0,01c	<0,01c	<0,01c	<0,01c
a. Test distribution is Normal.							
b. Calculated from data.							
c. Lilliefors Significance Correction.							

DNA and degradation index

For the patella, 45 bone segments were analysed. There were no missing (undetermined) values for the patella in the database.

For calcaneus, 104 bone segments were analysed. Four missing (undetermined) values for the amount of DNA in calcaneus bones were substituted with the value 0,04. There were 12 missing (undetermined values) for the degradation index of calcaneus bones in the database. According to Table Statistics Supplementary 1, undetermined values were substituted with the value 71,35.

For the talus, 60 bone segments were analysed. Six missing (undetermined) values for the amount of DNA in talus bones were substituted with the value 0,04. There were 16 missing (undetermined values) for the degradation index of talus bones in the database. Table Statistics Supplementary one shows that undetermined values were substituted with the value 47,7.

For navicular bone, 102 bone segments were analysed. Six missing (undetermined) values for the amount of DNA in navicular bones were substituted with the value 0,04. The database had 11 missing (undetermined values) for the degradation index of navicular bones. Table Statistics Supplementary one shows that undetermined values were substituted with the value 29,5.

For cuboid bone, 87 bone segments were analysed. One missing (undetermined) value for the amount of DNA in cuboid bones was substituted with the value 0,04. There were two missing (undetermined values) for the degradation index of cuboid bones in the database. According to Table Statistics Supplementary 1, undetermined values were substituted with 63,73.

For the medial cuneiform bone, 63 bone segments were analysed. There were no missing (undetermined) values for the amount of DNA in medial cuneiform bones. There was one missing (undetermined value) for the degradation index of medial cuneiform bones in the database. According to Table Statistics Supplementary 1, an undetermined value was substituted with the value 50,29.

Table Statistics Supplementary 3. Descriptive statistics for degradation index.

Degradation index		calcanues	talus	navicular bone	cuboid bone	medial cuneiform bone
N	Valid	92	44	91	85	62
	Missing	12	16	11	2	1
Std. Deviation		12,12	8,26	4,08	9,68	7,62
Maximum		59,23	39,44	25,42	54,05	42,67

Correlations between HU mean, DNA quantity and degradation index

Table Statistics Supplementary 4: Spearman's correlations for HU mean, DNA quantity and degradation index for the patella.

Spearman's coefficient with RUI (HU mean)	apex	anterior surface	posterior surface
Amount of DNA	0,17<br>(p = 0,55)	0,11<br>(p = 0,97)	-0,12<br>(p = 0,68)
Degradation index	-0,24<br>(p = 0,38)	0,58*<br>(p = 0,02)	0,14<br>(p = 0,63)

Table Statistics Supplementary 5: Spearman's correlations for HU mean, DNA quantity and degradation index, for calcaneus.

Spearman's coefficient with RUI (HU mean)	posterior process / calcaneal tuberosity	body	sulcus	anterior process
Amount of DNA	0,24<br>(p = 0,23)	0,10<br>(p = 0,62)	0,35<br>(p = 0,08)	0,49*<br>(p = 0,01)
Degradation index	-0,03<br>(p = 0,88)	0,26<br>(p = 0,20)	-0,23<br>(p = 0,26)	-0,31<br>(p = 0,12)

Table Statistics Supplementary 6: Spearman's correlations for HU mean, DNA quantity and degradation index, for talus.

Spearman's coefficient with RUI (HU mean)	head	sulcus	opposite side of talar B	trochlea	posterior calcaneal articular facet
Amount of DNA	-0,43<br>(p = 0,17)	0,83**<br>(p < 0,01)	0,75**<br>(p < 0,01)	0,64*<br>(p = 0,03)	0,55<br>(p = 0,06)
Degradation index	0,20<br>(p = 0,54)	-0,71**<br>(p < 0,01)	-0,84**<br>(p < 0,01)	-0,37<br>(p = 0,24)	-0,64*<br>(p = 0,03)

Table Statistics Supplementary 7: Spearman's correlations for HU mean, DNA quantity and degradation index, for the navicular bone.

Spearman's coefficient with RUI (HU mean)	proximal articular surface	distal articular surface	tuberosity
Amount of DNA	0,16 (p = 0,37)	0,17 (p = 0,34)	0,01 (p = 0,99)
Degradation index	-0,18 (p = 0,30)	-0,22 (p = 0,21)	-0,22 (p = 0,22)

Table Statistics Supplementary 8: Spearman's correlations for HU mean, DNA quantity and degradation index, for the cuboid bone.

Spearman's coefficient with RUI (HU mean)	proximal articular surface	tuberosity	distal articular surface
Amount of DNA	-0,06 (p = 0,77)	0,16 (p = 0,41)	0,01 (p = 0,96)
Degradation index	0,12 (p = 0,54)	-0,41* (p = 0,03)	-0,15 (p = 0,43)

Table Statistics Supplementary 9: Spearman's correlations for HU mean, DNA quantity and degradation index, for athe medial cuneiform bone.

Spearman's coefficient with RUI (HU mean)	proximal articular surface	medial surface	distal articular surface
Amount of DNA	-0,04 (p = 0,88)	-0,14 (p = 0,38)	-0,08 (p = 0,74)
Degradation index	-0,27 (p = 0,23)	0,05 (p = 0,77)	-0,23 (p = 0,33)

Correlations between HU mean, DNA quantity and degradation index according to bone type

Table Statistics Supplementary 10: Spearman's correlations for HU mean, DNA quantity and degradation index according to bone type, for the patella.

**Spearman's coefficient with RUI (HU mean)**	**spongy (cancellous bone)**	**compact (cortical bone)**
**Amount of DNA**	-0,12 (p = 0,68)	0,19 (p = 0,32)
**Degradation index**	0,14 (p = 0,63)	0,14 (p = 0,47)

Table Statistics Supplementary 11: Spearman's correlations for HU mean, DNA quantity and degradation index according to bone type, for calcaneus.

Spearman's coefficient with RUI (HU mean)	spongy (cancellous bone)	compact (cortical bone)
**Amount of DNA**	0,10 (p = 0,62)	**0,39** (p < 0,01)**
**Degradation index**	0,26 (p = 0,20)	**-0,23* (p < 0,05)**

Table Statistics Supplementary 12: Spearman's correlations for HU mean, DNA quantity and degradation index according to bone type, for talus.

Spearman's coefficient with RUI (HU mean)	spongy (cancellous bone)	compact (cortical bone)
**Amount of DNA**	0,07 (p = 0,74)	**0,64** (p < 0,01)**
**Degradation index**	0,06 (p = 0,77)	**-0,66** (p < 0,01)**

Table Statistics Supplementary 13: Spearman's correlations for HU mean, DNA quantity and degradation index according to bone type, for the navicular bone.

Spearman's coefficient with RUI (HU mean)	spongy (cancellous bone)	compact (cortical bone)
**Amount of DNA**	0,01 (p = 0,99)	0,21 (p = 0,09)
**Degradation index**	-0,22 (p = 0,22)	-0,23 (p = 0,07)

Table Statistics Supplementary 14: Spearman's correlations for HU mean, DNA quantity and degradation index according to bone type, for the cuboid bone.

Spearman's coefficient with RUI (HU mean)	spongy (cancellous bone)	compact (cortical bone)
**Amount of DNA**	0,01 (p = 0,96)	-0,04 (p = 0,75)
**Degradation index**	0,15 (p = 0,43)	-0,17 (p = 0,20)

Table Statistics Supplementary 15: Spearman's correlations for HU mean, DNA quantity and degradation index according to bone type, for the medial cuneiform bone.

Spearman's coefficient with RUI (HU mean)	spongy (cancellous bone)	compact (cortical bone)
**Amount of DNA**	-0,08 (p = 0,74)	-0,14 (p = 0,38)
**Degradation index**	-0,23 (p = 0,33)	0,05 (p = 0,77)
